# ELEVATED PREVALENCE AND RISK OF KIDNEY DISEASE AMONG SEXUAL MINORITY OLDER ADULTS IN THE US

**DOI:** 10.1093/geroni/igad104.2092

**Published:** 2023-12-21

**Authors:** Sean Cahill, Meghana Chandra, Mollie Hertel, Saumya Khanna, Shimontini Mitra, Jack Mirabella, Avareena Cropper

**Affiliations:** The Fenway Institute, Boston, Massachusetts, United States; NORC at the University of Chicago, Chicago, Illinois, United States; NORC at the University of Chicago, Chicago, Illinois, United States; NORC at the University of Chicago, Chicago, Illinois, United States; Fenway Health, Boston, Massachusetts, United States; Centers for Medicare & Medicaid Services, Baltimore, Maryland, United States; Centers for Medicare and Medicaid Services, Woodlawn, Maryland, United States

## Abstract

Pooled data from the Behavioral Risk Factor Surveillance System (2014–2019) were used for 22,114 lesbian, gay, bisexual adults and adults who said that their sexual orientation was “other” or “something else” aged 50 and over, and 748,963 heterosexual older adults. Logistic regressions were used to compare prevalence of self-reported kidney disease among older adults by sexual orientation. After adjusting for age, race and ethnicity, and BMI, GB+ men had increased odds of self-reported kidney disease (AOR 1.42; 95% CI, 1.21–1.68). LGB+ women also had higher odds of self-reported kidney disease (AOR 1.29; 95% CI, 1.09–1.52). After controlling for sociodemographic factors, health behaviors, access to healthcare, and self-reported coronary heart disease, HIV, and diabetes, older gay, bisexual and other men (GB+) men (AOR 1.3; 95% CI, 1.09-1.54) were more likely than their heterosexual counterparts to report kidney disease. LGB+ men and women also reported high incidences of known risk factors for CKD. For example, both GB+ men (OR 1.39; [95% CI], 1.26-1.54) and LGB+ women (OR 1.39; [95% CI], 1.25-1.55) were more likely to be smokers and have a higher incidence of activity limitations, adverse health outcomes, and limited access to health care, housing and employment. We did not find any statistically significant differences in kidney disease between transgender and cisgender respondents. There is an increased need for screening for CKD risk factors, for preventative education and culturally responsive and affirming care, and for interventions to address social drivers of marginalization and vulnerability in older LGBT+ adults.

